# Hartmann pouch herniation in Calot’s triangle: A case report

**DOI:** 10.1016/j.ijscr.2020.05.022

**Published:** 2020-05-22

**Authors:** Tariq Almnaizel, Tawfiq Alnawafleh, Ra’ed Al-Jarrah, Abdulhamid M. Al-Abadi, Malek A. Al-Omari, Eman A. Al-Oudat

**Affiliations:** a Department of General Surgery, King Hussein Medical Center (KHMC), Amman, Jordan; b Department of Pulmonology, King Hussein Medical Center (KHMC), Amman, Jordan

**Keywords:** Hartmann pouch, Calot’s triangle, Laparoscopic cholecystectomy

## Abstract

**Introduction:**

Laparoscopic cholecystectomy is one of the most frequent operations performed around the world. Some pathological findings are particularly rare and difficult to diagnose preoperatively. Here, we report a case of a patient who presented to our hospital with calculus cholecystitis with a unique Intraoperative finding of Hartmann pouch herniation through hepatocystic triangle. The aim of the study is to consider Hartmann pouch herniation as a rare differential diagnosis of gallbladder stone complication.

**Presentation of case:**

We present a 48-year-old male who came to our emergency department complaining of constant epigastric abdominal pain lasting 3 h with vomiting. Utilizing chemistry laboratory studies and radiological studies, the final diagnosis was acute calculus cholecystitis. Early laparoscopic cholecystectomy was done and revealed Hartmann pouch herniation through the Calot’s triangle. This herniation resulted in strangulation of the Hartmann pouch and displacement of the cystic duct and artery anteriorly.

**Discussion:**

The biliary tract is liable for congenital anomalies. These anatomical variations can be diagnosed either intra-operatively or pre-operatively using radiological imaging. Hartmann pouch herniation is a new finding that we encountered in this case.

**Conclusion:**

Profound surgeons’ anatomical knowledge is essential for the safety of patients, especially for hepatobiliary surgeries due to the wide variations in normal and pathological anatomy. Using critical view of safety can decrease avoidable complications. Consulting specialized hepatobiliary surgeon is preferred when dealing with such cases.

## Highlights

• There are many atypical anatomic structural variations of the biliary tree.
• The anatomical variations can be diagnosed either intra-operatively or pre-operatively.
• Surgeon’s experience and knowledge are essential to form the best surgical decision.
• A second opinion of a hepatobiliary surgeon decreases avoidable complex injury to biliary tract in selective cases.
• Fine handling of hepatocystic triangle and skeletonization of porta hepatis are the main principles of surgery.

## 1 Introduction

Hartmann’s pouch is a diverticulum that can occur at the neck of the gall bladder. It is one of the rarest congenital anomalies of the gall bladder [1]. Hartmann’s gallbladder pouch is a frequent but inconstant feature of normal and pathologic human gallbladders. There is a significant association between the presence of Hartmann’s pouch and gallbladder stones. Hartmann’s pouch is caused by adhesions between the cystic duct and the neck of the gallbladder. As a result, it is classified as a morphologic rather than an anatomic entity [2]. Here, we present the case of a patient who presented to our institution with abdominal pain. Clinical and radiological investigations suggested calculus cholecystitis, but laparoscopic cholecystectomy revealed herniation of the Hartmann pouch through Calot’s triangle, which is a peculiar finding. This work is submitted in line with the SCARE criteria [3].

## 2 Case presentation

A 48-year-old male presented to our emergency department complaining of constant epigastric abdominal pain lasting 3 h and radiating to the back. The pain started after a meal and was associated with vomiting. He reported similar attacks over the following few days. The patient was known to have hypertriglyceridemia. He had a history of appendectomy and hemithyroidectomy for a follicular lesion. Upon General examination, the patient showed normal vital signs, and no jaundice. Abdominal examination revealed a soft and lax abdomen with right upper quadrant and epigastric tenderness.

Laboratory tests revealed a WBCs of 7.2 cells/mm^3^, total bilirubin level of 0.55 mg/dl and serum amylase of 174 units/L. However, all liver enzymes and other electrolytes were within normal range. Abdominal ultrasonography showed signs of acute cholecystitis with a solitary large gallbladder stone impacted at the neck of the gallbladder. The final diagnosis was calculus cholecystitis. We admitted the patient and scheduled him for surgery. In the meantime, we kept him fasting and administered intravenous fluids and analgesics.

**Fig. 1:**
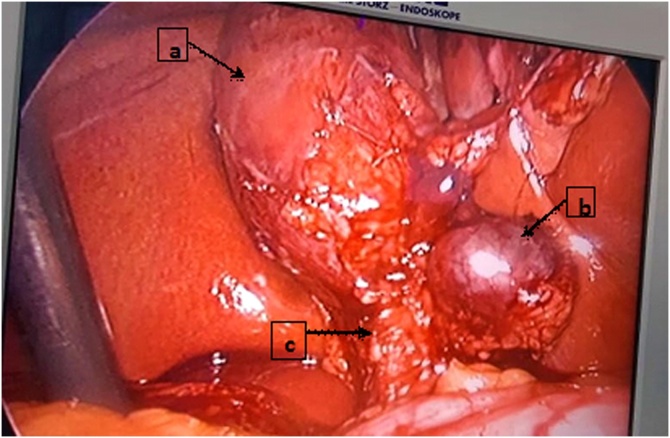
Image of the gallbladder after cephalad retraction, with careful dissection of the peritoneum covering the gallbladder. a, gallbladder; b, strangulated Hartmann’s pouch in the Calot’s triangle; c, cystic duct and artery.

**Fig. 2:**
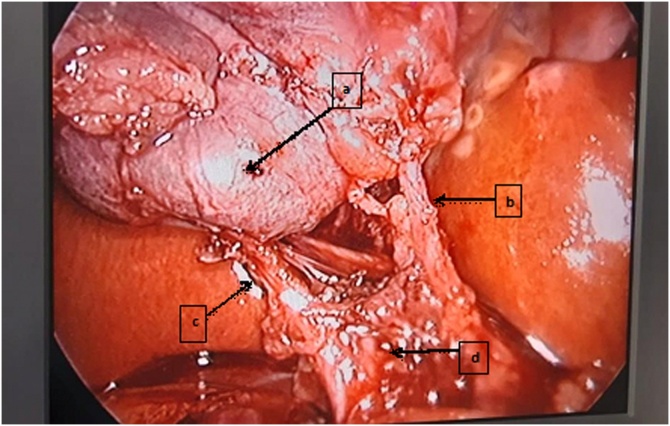
Calot’s triangle after careful dissection and release of the incarcerated Hartmann pouch creating critical view of safety (CVS). a, gallbladder; b, cystic artery; c, cystic duct; d, common hepatic duct.

After obtaining a consent form from the patient, laparoscopic cholecystectomy was planned for the next day. During the surgery, a bulging mass was observed medially in Calot’s triangle. It was hyperemic and incarcerated (Fig. 1). After careful dissection of Calot’s triangle, we found that this bulging mass continued to the neck of the gallbladder, displacing the cystic duct and artery anteriorly. After completing the dissection, we discovered that this bulging mass was actually a Hartmann pouch containing the gallbladder stone, which was herniated and incarcerated in the Calot’s triangle (Fig. 2). The procedure was completed laparoscopically, by releasing the Hartmann pouch from the Calot’s triangle, following the critical view of safety (CVS) protocol. The patient was discharged the next day. The postoperative period was uneventful.

## 3 Discussion

Cholecystectomy is the most common intra-abdominal surgical procedure performed in the United States. The laparoscopic approach is now the procedure of choice when cholecystectomy is indicated [4].

Hartmann’s pouch is named after Henri Albert Hartmann, who first described this rare diverticulum where the wall of the gallbladder neck joins the cystic duct. This cannot be a normal finding and is associated with an underlying pathology in all cases. It may also represent a site of gallstone impaction. The size of the pouch is variable. A larger Hartmann’s pouch may obscure the cystic duct and Calot’s triangle [8]. This represents a challenge for surgeons when performing Calot’s triangle dissection as it hinders correct identification of biliary and vascular anatomy.

Calot’s triangle was originally described by Calot in 1891 as an anatomical space bordered by the cystic duct, the bile duct and the cystic artery. In its present interpretation, the upper border is formed by the inferior surface of the liver, with the other two boundaries being the cystic duct and the bile duct. It usually contains the RHA, the cystic artery, the cystic lymph node (of Lund), connective tissue and lymphatic tissue. Occasionally, it may also contain accessory hepatic ducts and arteries [5]. A rare variant of the Calot’s triangle is the presence of five arteries, namely the RHA with its hepatic branch, and the CA with its two bifurcating branches. In a study on cadavers, the portal vein was observed within the Calot’s triangle in 15% (3/20) of cadavers. In one of the corpses, the portal vein and CA were present in this space, whereas the second corpse contained the portal vein and the RHA, and the third body contained only the portal vein in the triangle [9].

In cases of moderate-to-severe acute cholecystitis, inflammation can result in changes that may obscure the usual anatomical location or appearance of the vascular and biliary structures, including the cystic artery and the cystic duct. An ‘infundibular cystic duct,’ otherwise known as ‘hidden cystic duct syndrome’, can occur in acute cholecystitis as the cystic duct may be obscured by inflammation, leading the surgeon to misidentify the common bile duct as the cystic duct [6].

Hartman’s pouch and the gallbladder neck can sometimes be unexpectedly located beneath the common hepatic duct. This can lead the surgeon into falsely assuming that the common bile duct or the common hepatic duct is the cystic duct [6]. Furthermore, the neck of the gallbladder makes an angle with the fundus and creates the Hartmann pouch, which may obscure the common hepatic duct. This represents a potential crisis during cholecystectomy [7].

Findings such as those in clinical practice represent a challenge to the surgeon and a risk to the patient. The atypical anatomical findings demand rapid response and require proper conduct at the time of surgery to avoid complications. To our knowledge, this is the first case involving a Hartman pouch herniation and incarceration in Calot’s triangle reported in the literature.

## 4 Conclusions

Herniation and incarceration of Hartmann’s pouch in Calot’s triangle is one of the rarest pathological variations involving the content of the Calot’s triangle. After reviewing the literature, this appears to be the first case report with this finding. Understanding the variations of biliary anatomy remains key for performing safe and successful surgeries.

## Funding

This study has no sponsors and is self-funded.

## Ethical approval

Ethical approval has been taken from the ethical committe and King Hussein Medical center, Amman, Jordan. The reference number is 12/3-2020.

## Consent

Written informed consent was obtained from the patient for publication of this case report and accompanying images. A copy of the written consent is available for review by the Editor-in-Chief of this journal on request.

## Author contribution

Tariq Almnaizel MD: Main surgeon, concept development, investigation and supervision of findings.

Tawfiq Alnawafleh MD: assistant surgeon, drafting of article.

Ra’ed Al-Jarrah MD: assistant surgeon, data collection.

Abdulhamid M. Al-Abadi MD: data collection and analysis, writing of manuscript.

Malek A. Al-Omari MD: data analysis and interpretation, writing of manuscript.

Eman A. Al-Oudat RN: critical revision.

## Registration of research studies

Does not need registration.

## Guarantor

Tariq Almnaizel MD.

## Provenance and peer review

Not commissioned, externally peer-reviewed.

## Declaration of Competing Interest

The authors declare no conflicts of interests.
